# Acute spinal cord infarction secondary to ankylosing spondylitis: a case report and literature review

**DOI:** 10.3389/fneur.2023.1221810

**Published:** 2023-09-22

**Authors:** Wenjuan Li, Jia Guo, Lei Wang, Tinghua Zhang, Tian Li

**Affiliations:** ^1^Department of Neurology, Lanzhou University Second Hospital, Lanzhou, China; ^2^School of Basic Medicine, Fourth Military Medical University, Xi’an, China

**Keywords:** spinal cord infarction, ankylosing spondylitis, autoimmune disease, Taylor’s approach, vascular disease

## Abstract

**Introduction:**

Spinal cord infarction secondary to ankylosing spondylitis is a rare but severe disorder.

**Case presentation:**

Here we present a case of acute spinal cord infarction in a 54 years-old man with a medical history of ankylosing spondylitis, scoliosis, and hypotension. The patient complained of a sudden onset of lower limb weakness. A physical examination showed that he suffered from a dissociative sensory disorder, paralysis, and concomitant sphincter disturbances. After undergoing a whole-spine MRI, he was diagnosed with an acute ischemic injury from T2 to T5. As he did not treat his ankylosing spondylitis, it later caused a spinal deformity, making the lumbar puncture technically challenging. However, using Taylor’s approach, a CSF sample was successfully obtained. A CSF biochemical test ruled out myelitis, NMOSD, and MS. After receiving treatment with low-molecular-weight heparin, atorvastatin calcium, and methylprednisolone, his sphincter function gradually recovered, but his strength was only partially restored.

**Conclusion:**

Although this is a rare entity, it is necessary for physicians to consider it when evaluating patients with a sudden loss of sensation and strength in their lower limbs.

## 1. Introduction

Spinal cord infarction (SCI) is less frequent than ischemic brain injury and represents only approximately 1% of all strokes. It also accounts for 8% of myelopathies (1, 2). It can leave patients with devastating neurological sequelae such as paraplegia, quadriplegia, and incontinence. There are usually no obvious inducing factors, the rate of misdiagnosis is high, and there is no unified treatment plan. In general, the common etiologies of SCI include atherosclerosis, embolism, vertebral dissection, fibrocartilaginous embolism, hypercoagulable states, vasculitis, vascular malformations, and vascular compression. Additionally, systemic conditions such as cardiovascular disease, hypercoagulability, and hypotension have common etiologies. However, approximately one-third of SCIs have no identifiable etiology. Therefore, this is considered an idiopathic or cryptogenic disease. The progression of acute SCI is slower than that of cerebral infarction, peaking within 12 h and developing for up to 72 h (3). We treated a patient with a years-long history of ankylosing spondylitis who suddenly developed weakness in both lower extremities.

Common neurological complications of ankylosing spondylitis (AS) include stroke, cauda equina syndrome, and peripheral neuropathy (4, 5). However, AS associated with early-onset atherosclerosis has attracted the attention of doctors. Studies have shown that, compared with healthy people, patients with AS have a higher incidence of cardiovascular and cerebrovascular disease and mortality (6). They are more often associated with hypertension, metabolic syndrome, diabetes, and other risk factors.

Nevertheless, only a few cases of AS with SCI have been reported. The case we present may provide new insights into the possibility of a relationship between AS and SCI, whether secondary or combined.

## 2. Case presentation

A 54 years-old right-handed man was admitted to the Department of Neurology of The Second Hospital of Lanzhou University in September 2021 with a sudden onset of lower limb weakness. Four days before admission, the patient felt weakness in both lower limbs, more severe in the left leg, and was unable to walk. Two days later, the weakness in the right lower limb worsened, and he was unable to stand. He also presented with back pain, urinary retention, and constipation. The patient was a non-smoker with a medical history of scoliosis, hypotension, and AS, all of which were untreated. On examination, the subject’s blood pressure was 75/50 mmHg. At that time, his weight was 40 kg, and his spine was deformed and curved. The neurological examination revealed that his speech, cognition, and cranial nerve function were normal. He had full strength bilaterally in all muscle groups in his upper limbs, with bilaterally brisk reflexes and normal muscle tone. Conversely, in his lower limbs, his muscle tone was significantly decreased, with tendon hyporeflexia. In both lower limbs, he had a flaccid tone with a Medical Research Council (MRC) grade 1/5 strength in the left leg and an MRC grade 2/5 strength in the right leg. He had dissociated sensory loss, and his superficial sensation was lost, with relative sparing of deep sensation below the level of the Th6 dermatome. Bilateral Babinski signs were present. The modified Rankin scale (MRS) score was 4/5.

Laboratory tests showed: C-reactive protein (CRP) 55.54 mg/L (normal range <6 mg/L), erythrocyte sedimentation rate (ESR) 23 mm/h (normal range <20 mm/h), immunoglobulin A (IgA) 4.5 g/L (normal range <4 g/L), D-dimer 1.13 mg/mL (normal range <0.5 mg/mL), γ-glutamyl transpeptidase (γ-GT) 268 u/L (normal range 10–60 u/L), alkaline phosphatase (ALP) 205 u/L (normal range 45–125 u/L), triglycerides (TG) 4.44 mmol/L, lipoprotein (a) [Lp(a)] 482 mg/L (normal range <400 mg/L), creatine kinase (CK) 365 u/L (normal range 50–310 u/L); HLA-B27 was positive. Homocysteine (HCY), hemoglobin A1c (HbA1c), antiphospholipid antibodies, thyroid function, and routine anticoagulant blood tests were normal. Electromyography (EMG) showed multiple sensory and motor nerve damages. A color Doppler ultrasound showed a thrombus in the intermuscular vein of the left lower limb.

**Figure 1:**
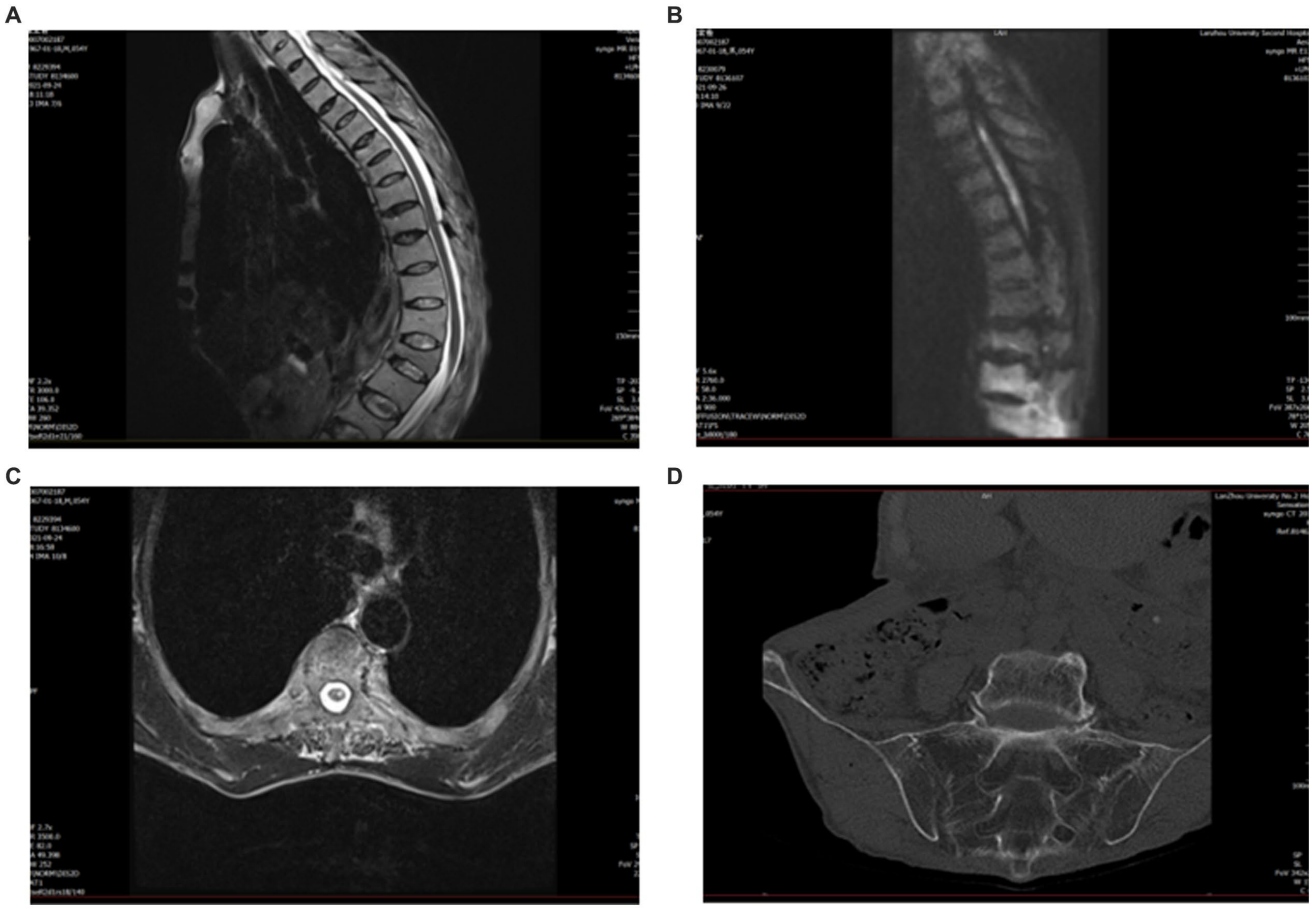
Pre-treatment magnetic resonance imaging (MRI). **(A)** Sagittal T2 cervical spine shows high signal T2–T5. **(B)** Diffusion-weighted imaging (DWI) shows restricted diffusion within the gray matter. **(C)** Axial T2 suggests a high signal centrally in the spinal cord. **(D)** CT of the sacroiliac joint shows bilateral sacroiliitis and calcification of the anterior and posterior longitudinal ligaments.

An MRI of the whole spine was undertaken. On the sagittal T2, there was an abnormal intramedullary signal intensity from T2 to T5, with central cord swelling. Diffusion-weighted imaging (DWI) with an apparent diffusion coefficient showed restricted diffusion, which can be seen in patients with acute ischemia (Figures 1A–C). A spinal angiogram was recommended but declined by the patient.

**Figure 2:**
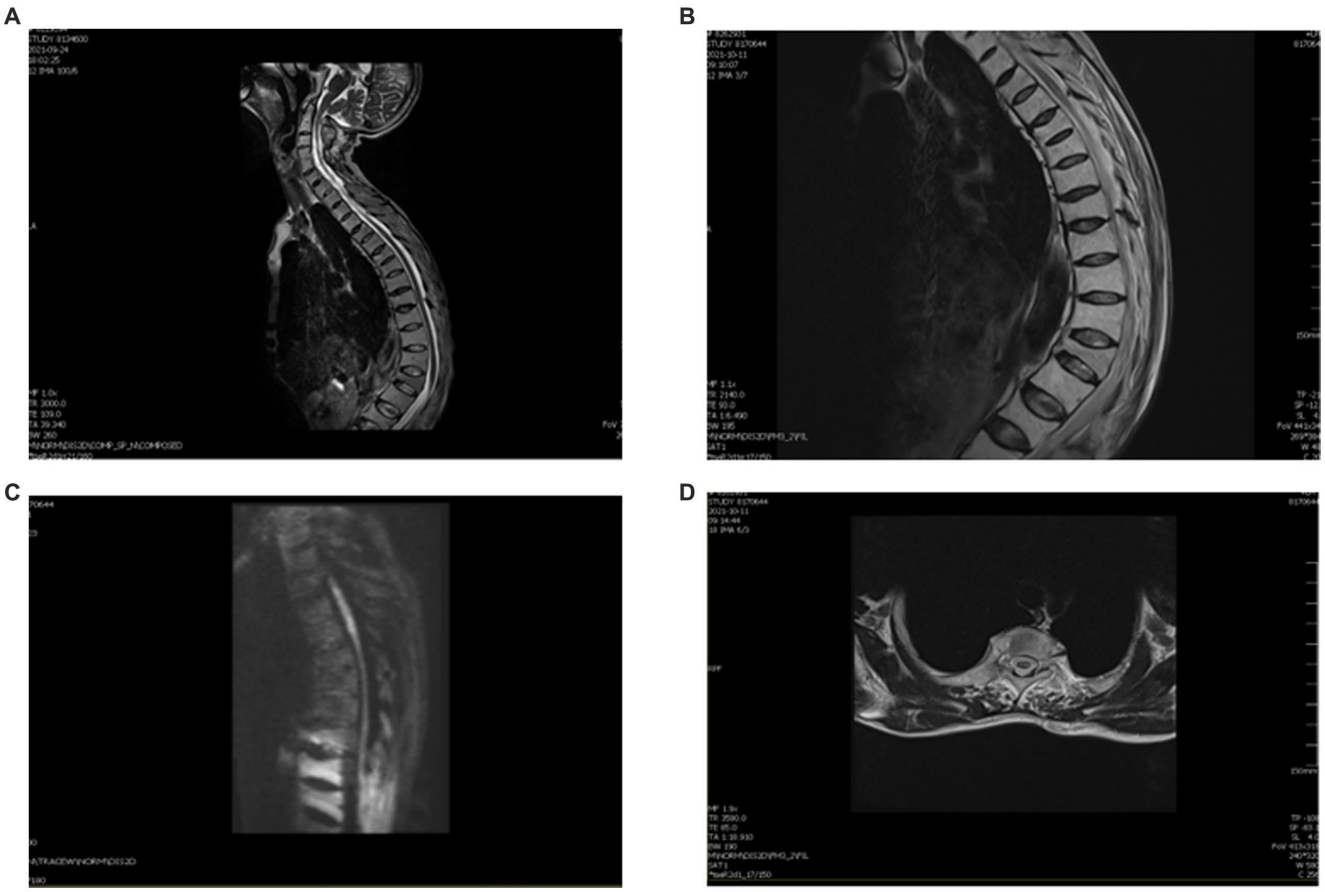
MRI of this patient after therapy. **(A)** Thoracic kyphosis with multiple vertebral wedgies at T4, T6, T7, T9, L1, and L3. **(B)** Sagittal T2 thoracic vertebrae T2–T5 are slightly hypersignal. **(C)** DWI shows a slightly elevated signal in the gray matter. **(D)** Axial T2 central hypersignal is less than before.

Due to his AS, the patient had spinal deformities, ligament ossification, and spinal space occlusion (Figure 2A), which made lumbar puncture technically challenging. CT of the sacroiliac joint showed that he had bilateral sacroiliitis and calcification of the anterior and posterior longitudinal ligaments (Figure 1D). A chest CT showed abnormalities of the thoracic vertebrae and an old fracture of the ribs. Experienced anesthesiologists performed multiple lumbar puncture attempts in the left lateral position at different levels (L2_3 and L3_4), with both midline and paramedian approaches, but these failed. After five failed attempts, the Taylor approach was successfully used for lumbar punctures. After local anesthetic infiltration, a spinal needle was inserted 1 cm medial and 1 cm caudal to the posterior superior iliac spine, which was located immediately in front of the skin depression. On the first attempt, a needle was inserted toward the medial side of the head toward the L5_S1 space to obtain clear cerebrospinal fluid. Cerebrospinal fluid analysis showed a normal cell count (3 m/L) and an elevated protein level (0.72 g/L). The patient was negative for serum anti-aquaporin4 antibody (AQP4), oligoclonal band (OB), and glial fibrillary acidic protein (GFAP).

An MRI was performed to differentiate between possible inflammation and infarction. The MRI demonstrated restricted diffusion from T2 to T5. Combined with a severe acute deficit within hours, the subject was diagnosed with SCI.

**Figure 3:**
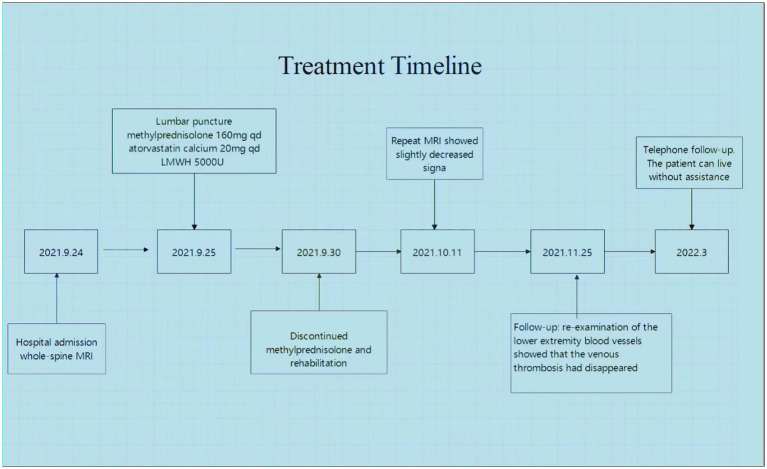
Treatment timeline of the patient.

We treated the patient with low molecular weight heparin (LMWH) for 1 month at the therapeutic dose, atorvastatin calcium (20 mg daily), and 160 mg intravenous methylprednisolone for 5 days, to reduce spinal cord edema. Non-steroidal anti-inflammatory drugs (NSAIDs) were prescribed for the symptomatic treatment of AS. After 2 weeks, a thoracic spine MRI showed a slightly decreased signal (Figures 2B–D). One month later, a reexamination of the lower vascular ultrasound showed unobstructed blood flow and resolution of venous thrombosis. After 8 weeks, the patient could walk with a medical walker. His MRS score was 3/5. A total of 6 months after the onset of his symptoms, he was able to walk independently and live without assistance. The treatment timeline is shown in Figure 3.

## 3. Discussion

Here we describe a case of acute SCI with a history of AS. The pathogenesis of acute SCI included aortic and venous pathology. In turn, the aortic pathology included mechanical radicular artery injury and spinal hypoperfusion. In a study of 131 patients with spontaneous spinal cord infarction (3), the suspected mechanism of SCI was idiopathic with atherosclerotic risk factors in 91 subjects (68%); fibrocartilaginous embolism, 19 (14%); aortic dissection, seven (5%); hypercoagulability, five (4%); vertebral artery dissection, four (3%); systemic hypotension, three (2%); cardioembolic, two (2%); and vasculitis, two (2%). Unfortunately, the patient refused to undergo a examination of spinal angiography. Thus, there is no direct examination results of the patient’s vascular condition. The patient had a long history of hypotension. Combined with the patient’s frail body habitus, we believe that his current blood pressure level was relatively normal for him. Additionally, there were no further apparent induced incidents such as diarrhea, reduced diet, or sweating, before the onset of this spinal infarction. Even though hypotension was probably not the main cause of infarction in this patient, it could be a significant contributing factor in combination with AS and radicular artery compression.

Ankylosing spondylitis is a chronic, progressive autoimmune rheumatic inflammatory disease. Patients with AS have an elevated risk of developing cerebrovascular disease. These factors include continuous systemic inflammation and lipid metabolism disorders (7). Chronic systemic inflammation may contribute to endothelial damage, diastolic dysfunction, and accelerated atherosclerosis. It may also lead to small-vessel inflammation. There are reports (8) suggesting that patients with AS have significantly higher levels of TG, HDL, and Lp(a), but these are potential contributing factors. Thus, patients with AS exhibit lipid metabolism disorders that work together with chronic systemic inflammation in the process of atherosclerosis. Our patient’s high levels of CRP and ESR may indicate that his AS was active, although this is not definitive. His lipid levels [TG, LDL, and Lp (a)] were all high, suggesting that he had only lipid disorders.

This patient’s EMG suggested peripheral neuropathy. This could be a complication of AS. There are probably several different mechanisms, such as demyelination, disruption of the blood supply (probably of arterial origin), and areas of arachnoiditis occurring earlier in the disease and resulting in atrophy. The latter is possible because patients usually develop their symptoms over a few months and then do not progress further (9).

There have been reports of cardiovascular disease associated with AS, and the pathogenesis of cardiovascular and cerebrovascular disease is similar, allowing the pathogenesis of spinal cord vascular disease. The related mechanisms include the following aspects: first, there is secondary systemic vasculitis. Second, AS accelerates the atherosclerosis in myelopathy. Finally, even though the patient’s weight loss, chronic hypotension, chronic malnutrition, and other factors (e.g., chronic adrenal insufficiency in a chronically ill patient) may have contributed to spinal ischemia, AS was still the primary cause. Therefore, our patient was ultimately diagnosed with SCI secondary to AS.

Due to his kyphotic scoliosis, the conventional technique for lumbar puncture failed. Lumbar puncture with Taylor’s approach targeting the L5-S1 interlaminar space was performed as an alternative. The reason is that targeting the L5_S1 interlaminar space provides a reliable alternative to the midline approach for lumbar puncture that is least affected by arthritic and degenerative changes (10).

Unfortunately, there are no universally accepted and effective methods to treat SCI. A few case reports advocate using IV thrombolysis in SCI patients in the hyperacute period (11, 12). However, no public data are available to support their use. In practice, treatment is usually based on the etiology of spinal cord ischemia. If no clear etiology is found, risk factor modification with blood pressure and glucose control, statins, and antithrombotics is often considered. We treated the patient with methylprednisolone, aiming to reduce the spinal cord edema. Due to his high D-dimer and deep venous thrombosis, we used antithrombotic therapy. With the applied therapy, his blood pressure increased to 110/70 mmHg.

We searched the PubMed database using the search strategy “(spinal cord infarction) and (ankylosing spondylitis)” and found seven items, one of which was relevant. Shim et al. (13) described a man with ankylosing spondylitis who developed multiple cerebellar infarctions due to vertebral artery obstruction and bulbar symptoms associated with atlanto-occipital subluxation and vertical subluxation. Although there are few reports on the correlation between spinal cord infarction and ankylosing spondylitis, there are many reports on spinal cord infarction.

Isolated posterior spinal cord infarction is rare, and vertebral artery dissection should be considered as an etiologic mechanism. Montalvo et al. (14) described a patient with an acute onset of flaccid quadriplegia. He regained complete muscle strength within 1 h. His MRI of the cervical spine showed a C3–C4 level consistent with ischemic stroke. MR angiography showed a vertebral artery dissection. The extensive collateral vascular network perfusing the spinal cord makes the incidence of spinal infarction low. Gu et al. (15) reported an acute spinal cord infarction due to a type B intramural hematoma of the descending aorta. The patient was able to walk with an elbow crutch after 2 years.

Recently, Zalewski et al. (3) proposed diagnostic criteria for SCI. These emphasize three major components: (1) clinical: rapid development of severe deficits within 12 h; (2) MRI spine: exclusion of compression (2A) with supportive features (2B), specific imaging findings (2C); and (3) CSF: emphasis on noninflammatory findings in most cases.

## 4. Conclusion

We presented a rare case of SCI related to AS, which was both interesting and significant. It may help clinicians have a better understanding of the potential for ischemic myelopathy associated with AS.

## Data availability statement

The datasets presented in this article are not readily available because of ethical and privacy restrictions. Requests to access the datasets should be directed to the corresponding authors.

## Ethics statement

The studies involving humans were approved by The Ethics Committee of Lanzhou University Second Hospital. The studies were conducted in accordance with the local legislation and institutional requirements. The participants provided their written informed consent to participate in this study. Written informed consent was obtained from the individual(s) for the publication of any potentially identifiable images or data included in this article.

## Author contributions

JG, TL, and WL conducted the research, participated in data collection, and drafted the manuscript. JG, LW, WL, and TZ conceived and participated in the design. All authors contributed to the article and approved the submitted version.

## Figures and Tables

**Table 1:** 

SCI	Spinal cord infarction
AS	Ankylosing spondylitis
MS	Multiple sclerosis
NMOSD	Neuromyelitis optica spectrum disorder
MRC	Medical Research Council
MRS	Modified Rankin scale
CRP	C-reactive protein
ESR	Erythrocyte sedimentation rate
IgA	Immunoglobulin A
γ-GT	γ-glutamyl transpeptidase
ALP	Alkaline phosphatase
TG	Triglycerides
EMG	Electromyography
MRI	Magnetic resonance imaging
DWI	Diffusion-weighted imaging
AQP4	Anti-aquaporin 4 antibody
OB	Oligoclonal band
GFAP	Glial fibrillary acidic protein
LMWH	Low-molecular-weight heparin
